# Lime Peel Oil–Incorporated Rosin-Based Antimicrobial In Situ Forming Gel

**DOI:** 10.3390/gels8030169

**Published:** 2022-03-08

**Authors:** Ei Mon Khaing, Jongjan Mahadlek, Siriporn Okonogi, Thawatchai Phaechamud

**Affiliations:** 1 Programme of Pharmaceutical Engineering, Faculty of Pharmacy, Silpakorn University, Nakhon Pathom 73000, Thailand; eimonkhaing10@gmail.com; 2 Pharmaceutical Intelligence Unit Prachote Plengwittaya, Faculty of Pharmacy, Silpakorn University, Nakhon Pathom 73000, Thailand; mahadlek_j@su.ac.th; 3 Natural Bioactive and Material for Health Promotion and Drug Delivery System Group (NBM), Faculty of Pharmacy, Silpakorn University, Nakhon Pathom 73000, Thailand; 4 Research Center of Pharmaceutical Nanotechnology, Faculty of Pharmacy, Chiang Mai University, Chiang Mai 50200, Thailand; siriporn.okonogi@cmu.ac.th; 5 Department of Pharmaceutical Sciences, Faculty of Pharmacy, Chiang Mai University, Chiang Mai 50200, Thailand; 6 Department of Pharmaceutical Technology, Faculty of Pharmacy, Silpakorn University, Nakhon Pathom 73000, Thailand

**Keywords:** in situ forming gel, antimicrobial, rosin, lime peel oil

## Abstract

Localized intra-periodontal pocket drug delivery using an injectable in situ forming gel is an effective periodontitis treatment. The aqueous insoluble property of rosin is suitable for preparing a solvent exchange-induced in situ forming gel. This study aims to investigate the role of incorporating lime peel oil (LO) on the physicochemical properties of injectable in situ forming gels based on rosin loaded with 5% *w*/*w* doxycycline hyclate (DH) in dimethyl sulfoxide (DMSO) and *N*-methyl pyrrolidone (NMP). Their gel formation, viscosity, injectability, mechanical properties, wettability, drug release, and antimicrobial activities were evaluated. The presence of LO slowed gel formation due to the loose precipitate formation of gel with a high LO content. The viscosity and injectability were slightly increased with higher LO content for the DH-loaded rosin-based in situ forming gel. The addition of 10% LO lowered gel hardness with higher adhesion. LO incorporation promoted a higher drug release pattern than the no oil-added formulation over 10 days and the gel formation rate related to burst drug release. The drug release kinetics followed the non-Fickian diffusion mechanism for oil-added formulations. LO exhibited high antimicrobial activity against *Porphyromonas gingivalis* and *Staphylococcus aureus*. The DH-loaded rosin in situ forming gel with an addition of LO (0, 2.5, 5, and 10% *w*/*w*) inhibited all tested microorganisms. Adding 10% LO to rosin-based in situ forming gel improved the antimicrobial activities, especially for the *P. gingivalis* and *S. aureus.* As a result, the study demonstrates the possibility of using an LO amount of less than 10% loading into a rosin-based in situ forming gel for efficient periodontitis treatment.

## 1. Introduction

Periodontitis damages the periodontal tissue and destroys the bone that supports the teeth resulting in periodontal pockets for bacteria accumulation with more aggressive conditions [1]. In practice, the inhibition of bacteria growth in periodontal pockets by mechanical scaling, root planing and medications are typically recommended by the dentist [1,2]. Several antimicrobial agents have been utilized for clinical and microbiological efficacy in periodontal diseases such as chlorhexidine, metronidazole, ciprofloxacin, doxycycline, etc. [2]. However, they exhibit some limitations such as serious side effects, antibiotic resistance, local irritation and undesired burst drug release [3]. The tiny irregular shapes and various sizes of periodontal pockets seem to be the problem for solid drug delivery systems to be inserted for a good fit. Thus, the localized in situ forming drug delivery system, with the liquid form first, demonstrates benefits over those drug delivery systems due to fitting well into the pocket before solidifying, ease of use and acceptable clinical effectiveness with its highly effective drug concentration at the target site with minimal side effects [4].

The solvent exchange-based in situ forming gel system has grown in importance as an injectable drug delivery system because of its many applications. The in situ forming gel system is a solution before administration and is mainly based on a biodegradable polymer, biocompatible solvent, and active drug. When this solution is injected into the body, the water-miscible solvent diffuses into the surrounding tissue, allowing water to infiltrate the system, and phase separation occurs, inducing the formation of a semi-solid-like matter [5]. The incorporated drug is released over a period from the polymer precipitate through diffusion or erosion of the polymer. The main merit of this dosage form is its easy preparation, sustained release of the active drug for a prolonged period at the injection site and decreased side effects from the drugs [6,7,8]. Therefore, in situ forming gel formulations are considered helpful in treating chronic disorders like periodontitis. Atridox is a commercially marketed product of the in situ forming gel that contains D,L-lactic acid (PLA) and *N*-methyl-2-pyrrolidone (NMP) loaded with 5 or 10% *w*/*w* doxycycline hyclate (DH) for periodontitis treatment [9].

Different synthetic and natural materials have been investigated for use as the matrix-forming material for an in situ forming gel, such as sucrose acetate isobutyrate-polylactic acid (SAIB), shellac, cholesterol, and fatty acids [6,10,11,12]. These materials are aqueous insoluble and soluble in some biocompatible organic solvents, which can be used to form solvent exchange-induced in situ forming gel. Organic solvents such as NMP and DMSO are solvents commonly used to develop an in situ forming gel due to their water miscibility and biocompatibility [5,13]. Rosin is an interesting natural material obtained from pine trees, which is locally available, eco-friendly, biodegradable, and biocompatible [14]. In pharmaceutical drug delivery systems, rosin has been widely used for microencapsulation, film coating, and tablet formulations [15]. Lee et al. used rosin nanoparticles as drug carriers for hydrocortisone-controlled release [16]. The aqueous insoluble property of rosin makes it suitable for preparing a solvent exchange-induced in situ forming gel. It was previously reported that rosin in some organic solvents could transform from a solution into insoluble matter in an aqueous environment [17]. However, no additives were applied to the rosin-based in situ forming gel, and the effect of additives on the formulation qualities has not yet been determined.

It has been discovered that the addition of additives to the in situ forming gel influences drug release and adhesive properties. For example, the addition of oils such as peppermint and clove into Eudragit RS in situ forming gel has been investigated to decrease the burst release of DH [18,19]. Additionally, the stickiness of in situ implants was enhanced by adding plasticizers and bio-adhesive polymers [20]. Moreover, Bode et al. investigated whether the addition of different additives into in situ forming implants could change morphology, swelling kinetics, and drug release rate [21]. The results showed that hydrophilic additives caused an implant swelling, whereas lipophilic additives might promote a phase separation.

Lime peel oil (LO) has antioxidant, hypolipidemic, cytotoxic, and antimicrobial activities [22,23]. The most important compounds in LO are limonene, terpinene, and pinene. The volatile oils from citrus, including LO, exhibited the antimicrobial activities against Gram-positive/Gram-negative bacteria, yeast and mold [24]. The volatile oil-added pectin-based films could decrease the mechanical qualities and enhance antimicrobial properties [25]. Moreover, the combination of essential oils and antibiotic drugs could enhance the therapeutic effect of drug resistance strains [26]. Hence, in this research, there was a plan to incorporate additives such as LO into a rosin-based in situ forming gel to promote the antimicrobial activities. DH is an antibiotic that has been used for treating periodontitis. The DH-loaded polymer-based drug delivery systems have been formulated to sustain drug release in order to decrease the frequency of administration [27,28].

Thus, this study investigated the effect of LO incorporation on the physicochemical properties including viscosity, injectability, mechanical properties, wettability, drug release, and the antimicrobial characteristics of DH-loaded rosin-based in situ forming gels. Macroscopic and microscopic observations were also used to investigate the gel formation characteristics. 

## 2. Results and Discussion

### 2.1. Study of Gel Formation

#### 2.1.1. Gel Formation

**Figure 1:**
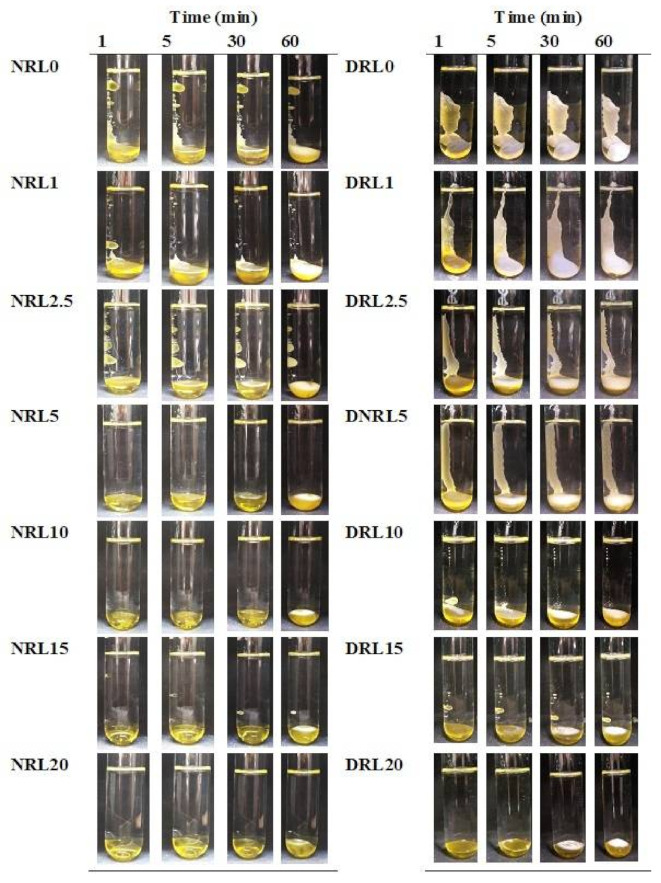
Gel formation of drug-free rosin-based in situ forming gel with various concentrations of LO using NMP and DMSO solvents in PBS pH 7.4 with different time intervals by visual observation. N = NMP, D = DMSO, R = rosin, L = lime peel oil.

All prepared formulations of DH-free rosin-based in situ forming gels with differing LO amounts (1–20% *w*/*w*) in NMP and DMSO were prepared in which all components completely dissolved after mixing overnight. DMSO and NMP were first used to prepare rosin in situ forming gel to select a suitable solvent for LO addition into the preparations. In situ forming gels were formed from solution to gel after injecting all formulations in the phosphate buffer solution (PBS) pH 7.4 (Figure 1). The test tubes were allowed to stand for one hour at room temperature to verify the gel formation appearance, and photos of the gel were taken at that time. The gel-forming process was influenced by the solvent used in the formulations. The formulations prepared with NMP were soft, transparent, and yellowish gels. The thickness of the gel-like substance could be observed as an opaque layer of surface gel at 30–60 min. The quick transformation from solution into gel was shown in DMSO-prepared formulations, which showed a more compact and thicker layer as time passed. This was possibly due to the water miscibility of the solvent, causing increased solvent exchange and then rapid gel formation. Bleached shellac-based in situ forming gel systems using DMSO as a solvent exhibited a faster transformation into gel than that of a system using NMP [29]. The use of DMSO as a solvent in the in situ forming gel formulation was proposed to allow for faster diffusion of the solvent exchange [10,30]. 

Moreover, gel transformation also depends on the addition of a hydrophobic substance. Adding more LO into the system also reduced the gel formation rate. The slower solvent exchange was observed when LO concentrations were increased from 5–20% in the NMP solvent and 10–20% in the DMSO solvent, resulting in a long time until a solid-like gel started to form. The incorporation of oil apparently interfered with the gel formation rate owing to lowering the miscibility of the components. The slow phase separation rate was observed when more clove oil was added to the lauric acid-based in situ forming gel due to the oil retarding water entering the system [31]. However, all formulations exhibited gelling as a thick opaque layer with time in the test tube. This phenomena could be attributed to the water insolubility characteristic of rosin that is utilized as a moisture-barrier coating agent due to its hydrophobicity [14,32].

#### 2.1.2. Microscopic Observation of Gel Formation

**Figure 2:**
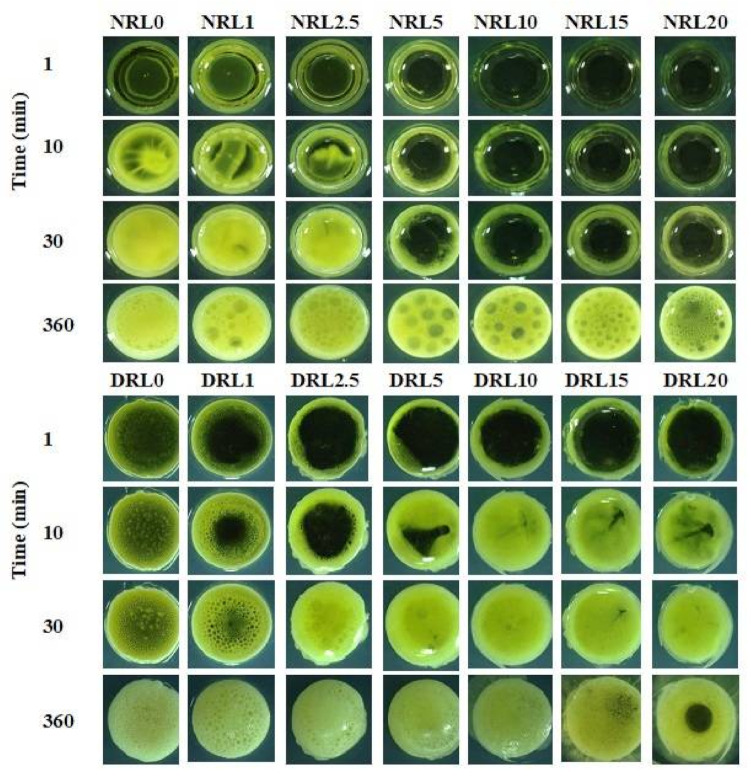
The gel formation of drug-free rosin-based in situ forming gel with various LO concentrations in agarose gel with different time intervals by microscopic observation using a stereomicroscope at magnification of ×100.

The top view of in situ forming gels was observed under a stereomicroscope for drug-free rosin-based in situ forming gel containing LO dissolved in NMP and DMSO that formed gel upon contact with PBS pH 7.4 in the agarose well (Figure 2). The results verified that gelling after contact with the aqueous from agarose of rosin containing LO in NMP took a longer time than that in DMSO when compared at the same percentage of oil used. All formula using DMSO as a solvent indicated an opaque surface layer within 30 min. Then slower solvent movement occurred with time and the opaque ring transitioned into a stable gel structure at 360 min. The solvent in the formulations diffused through the aqueous environment while the water diffused into the system at the early transition period [33]. The water-insoluble rosin became precipitated as fast as the diffusion of the solvent movement and contact with the rosin component. The gelling took more time as the amount of LO used was increased. For example, when the LO amount was increased from NRL5% to NRL20% *w*/*w*, the gelling time significantly increased from 30–360 min, especially for formulations prepared in NMP. The oil droplet on the gelling surface was found for all oil-loaded formulations after one hour. The hydrophobic nature of the oil-retarded water entering the gel reduced the rate of gel formation [34]. The slow phase inversion could affect the gel morphology and the release of loaded drugs [35]. The results were consistent with the previous macroscope observation of gel formation behavior of rosin-based in situ forming gel.

#### 2.1.3. Interfacial Phenomena

**Figure 3:**
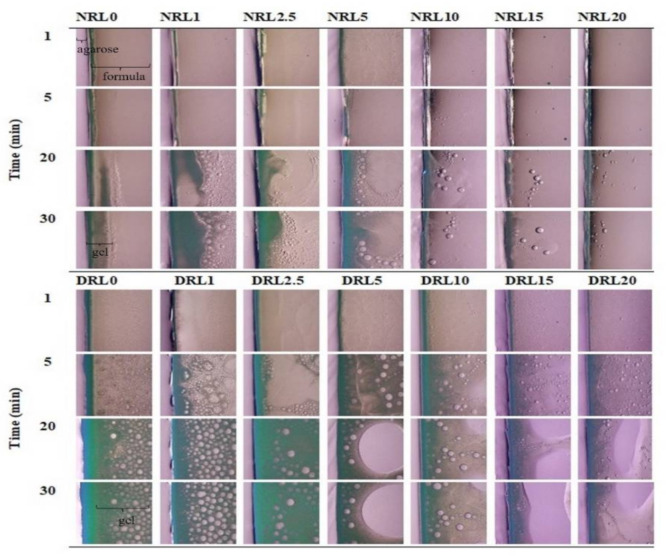
Interfacial phenomena changes of rosin in situ gel with different concentrations of LO after contact with aqueous agarose gel phase under stereomicroscope with various time intervals (40×).

Figure 3 shows the interface interaction under stereomicroscope. All formulations in the NMP took a longer time to form gel networks. The phase transformation into a gel network was evident at 20 min when the oil content was less than 5%. The obtained cloudy opaque greenish phase at 30 min (Figure 3 for NRL0-NRL5) resulted from the formation of a rosin gel during the solvent exchange between water and an organic solvent. The other formulations contained the oil droplets during phase separation. Formulations prepared in DMSO solvent exhibited gel formation at five min and became a denser gel of rosin with time. The gel structure changed from an emulsion-like appearance (Figure 3 DRL2.5, DRL5 and DRL10) to a loose precipitate formation of gel (Figure 3 for DRL15 and DRL20) when the LO concentration was increased. With increasing LO concentration 1–20%, water penetration into the system gradually decreased. LO addition might influence the gel formation by lowering the interface tension between the polymer and the solvent [36].

The appearance of the rosin gel was observed as the aggregation of small particles when the solvent exchange of DMSO and water occurred. In contrast, a rosin nucleation-like formation was found from the solvent exchange between NMP and water inducing rosin phase separation. The nucleation was generated at the interaction of two phases, inducing the agglomeration of particles [12,37]. These interfacial phenomena could indicate the microscopic self-forming behavior of the rosin/LO complex using various solvents. Understanding gel formation behavior is beneficial for estimating the drug release rate. The results showed that the formulations comprising LO content less than 10% in DMSO and 5% in NMP could be prepared in the in situ formation gel systems because of their dense gel formation. Nevertheless, the more rapid gel formation rosin/LO combinations prepared in DMSO were more appropriate than those prepared in NMP. 

### 2.2. Viscosity and Injectability

The viscosity and injectability of rosin-based in situ forming gel are presented in Table 1. Because of their quick gel-forming abilities, rosin in situ forming gels prepared in DMSO and LO concentrations of 0%, 2.5%, 5%, and 10% were chosen for the incorporation of 5%DH. The viscosity results indicated that the addition of LO decreased viscosity significantly (*p* < 0.05). All prepared drug-free formulations had viscosity values ranging from 122.60 to 191.40 cP. The formulations using NMP as the solvent had a higher viscosity than those in DMSO. These results supported the gel formation of formulations in NMP that took a longer gelling time because of their high viscosity values. The results showed that the rosin/LO combination dissolved better in DMSO because of the low viscosity of the solution. When a solvent with a high potential to dissolve polymers is used, lower viscosity solutions are obtained [12]. For example, in situ forming gel based on bleach shellac dissolved in DMSO obtained a low viscosity when compared to that of in NMP and 2-pyrrolidone [29].

The drug-loaded formulations had a significantly higher viscosity than drug-free formulations (*p* < 0.05). The viscosities of the 5%DH-loaded rosin-based in situ forming gel, comprising LO (0%, 2.5%, 5%, and 10%), were in the range of 624.51–684.15 cP. The DHRL0 to DHRL5 presented no statistically significant difference in viscosity results (*p* ≥ 0.05). After drug incorporation, less solvent in the system made the preparation more viscous and increased viscosity [38]. The formulation viscosity was increased as the amount of LO increased owing to the replacement of less viscous DMSO with more viscous LO. Clove oil also increased the viscosity of Eudragit RS-based in situ forming gel [19]. However, the viscosity value was lower than other injectable thermo-responsive in situ forming gel systems such as carbopol–poloxamer gels, the viscosity values off which were in the range 19,000–36,000 cP at 4 °C [39,40]. 

The force and work of injectability of the formulations were high in the system using NMP as a solvent and tended to reduce significantly after LO addition due to the lubricating property of the oil (Table 1). The force of injectability of all prepared drug-free systems was less than 4 N using a 24G needle, indicating that in situ forming gel was easily injected at the application site. The force of injectability of the DH-loaded formulations were also significantly higher (*p* < 0.05) compared to drug-free formulations and corresponded with their viscosity values. The work and force for expelling the prepared solutions increased when increasing LO from 2.5–10%. However, all formulations could be injected easily with a force lower than 21 N through a 24G needle with a 1 mL syringe. It was reported that an injected force lower than 25 N was recorded as an easy administration for the injection of formulations [41]. 

### 2.3. Mechanical Properties

**Figure 4:**
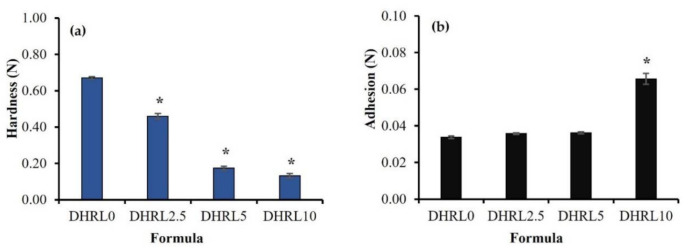
Mechanical properties of oil/rosin in situ forming gel after gel formation at three days: (**a**) hardness of gel, (**b**) adhesion of gel (n = 3). * Significant difference, *p* < 0.05.

Figure 4 indicates the mechanical properties of the DH-loaded formulations. The hardness of the rosin in situ forming gel with LO (0%, 2.5%, 5% and 10%) combination after complete gel formation at three days was 0.672 ± 0.007, 0.460 ± 0.015, 0.173 ± 0.011, and 0.132 ± 0.011 N, respectively (Figure 4a). The hardness of the rosin gel without LO was significantly higher than that of formulations with LO (*p* < 0.05) due to the complete solvent exchange promoting the harder precipitated rosin gel. The hardness was reduced with an increasing concentration of LO in the formulation. Increasing LO minimized the interaction forces of the rosin components, generating an inhomogeneous structure and lowering the firmness of the gel. The addition of oil reduced the film’s mechanical properties owing to the plasticizing effect of oil [42]. Actually, a formulation should not be too hard in order for it to deform and fit well with the periodontal pocket with time [8]. 

The adhesion of rosin in situ forming gels containing LO had the opposite effect on the hardness of the gel, and the results are indicated in Figure 4b. The adhesion of the gel increased from 0.0339 ± 0.001 N to 0.0657 ± 0.003 N with an increase of LO content from 0–10%. The LO additions of 2.5 and 5% did not much affect the adhesion of the gel. However, the addition of 10% LO affected the adhesion of the in situ forming gel, which significantly varied from other formulations (*p* < 0.05). The results show the benefits of using LO in rosin in situ forming gel formulation to increase adhesiveness. A previous study reported that the addition of 5% tea tree oil enhanced the adhesiveness of a hydrogel [43]. Typically, the adhesion of in situ forming gel could be improved by increasing the mucoadhesive polymer concentrations [40]. The higher gel adhesion provides greater stability and increases the retention of formulation at the application site [44,45]. 

### 2.4. Wettability of In Situ Forming Gel

**Figure 5:**
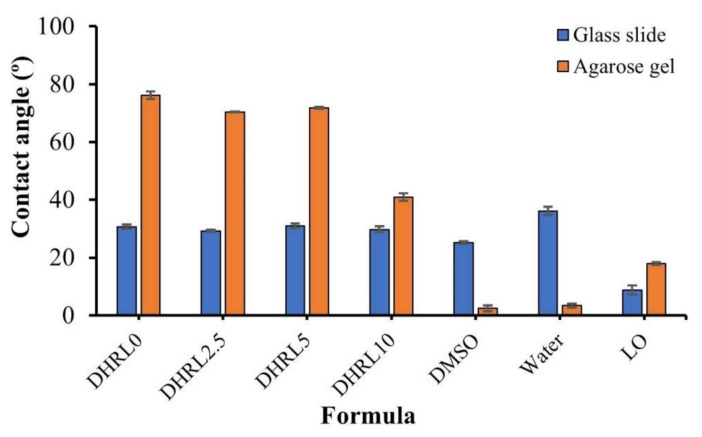
Wettability of rosin in situ forming gel on the surface of the glass slide and agarose gel (n = 3).

Contact angle measurements were used to determine the wettability of formulations between the formulation droplet and the surface. The formulations on the surface of agarose gel (simulated periodontal pocket tissue) showed a greater contact angle value than that of the surface of glass slides (Figure 5). This obvious higher contact angle value on agarose gel was due to gel formation from the phase separation of the formulation after contacting the aqueous phase of the agarose gel [46]. This transformation from solution into gel on agarose retarded the spreadability of the droplet. The contact angle values of rosin-based formulations on the agarose gel surface were significantly higher than those of LO, water, and DMSO, respectively. The tested formulations had similar wettability on both surfaces, whereas 10% LO addition indicated a significantly lower contact angle value on the surface of agarose gel. Therefore, the wettability related to the slow gel formation rate of the formulation on the agarose surface. Adding more oil to the formulations allowed for the reduction of the dense gel network, causing better spreading ability and a reduced contact angle. Nevertheless, all formulations showed a contact angle lower than 80°, showing good wettability, which promotes adhesiveness in the periodontal pocket. 

### 2.5. In Vitro Drug Release

**Figure 6:**
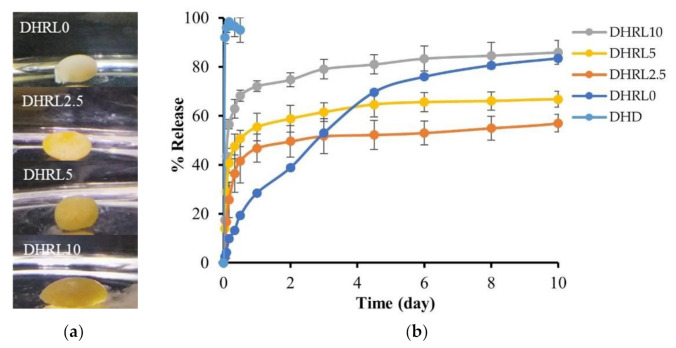
DH-loaded rosin in situ forming gel with various concentrations of LO: (**a**) Gel formation in the release medium after one hour; (**b**) DH release behavior on oil/rosin in situ forming gel over ten days (n = 3).

The DH-content in the drug-loaded formulations was in the range of 97.26 ± 1.72% to 102.44 ± 0.81%. All the tested formulations were observed to form a gel as soon as they contacted the release medium in Figure 6a. The direct contact method was employed to study the in vitro drug release instead of the dialysis tube method because the stickiness of the forming rosin gel had the potential to adhere to the dialysis tube surface which would disturb solvent/drug migration. The release profiles of DH from the rosin-based systems with and without LO addition are indicated in Figure 6b. A relationship was found between the rate of gel formation and the drug release rate. The DH release profile from LO-free formulations (DHRL0) was different from that of LO-loaded formulations. It indicated a low initial burst release (29% on the first day) and continuous release up to 80% for ten days. Because of the fast diffusion of solvent and water, there was no barrier from the oil when diffusing, resulting in rapid gel formation. As a result, the initial drug release phase was slower, and the prolonged-release lasted up to ten days.

The LO-loaded (2.5%, 5%, and 10%) formulations revealed an initial burst release followed by stable drug release for ten days. The drug release patterns of the 2.5% and 5% LO-incorporated in situ forming gel exhibited an initial release of 40% and 56%, respectively. After injection into the release medium, the gel shape of these two formulations was a soft, sphere-like gel (Figure 6a). The 10% LO-loaded gel exhibited faster DH release, nearly 70% on the first day, than the other formulations. The shape of the gel for this formulation showed an oval shape as a high concentration of LO content was added because LO reduced the hardness of the gel as described in the mechanical investigation (Figure 4a). Thus, as the LO content increased, the initial burst of drug release also increased.

Typically, adding a hydrophobic substance reduces the release rate of loaded drugs from controlled drug delivery systems. According to a previous study, incorporating hydrophobic oil into the in situ forming gel reduced the drug release rate [18,19]. The addition of clove oil to the Eudragit RS-based in situ forming gel reduced the release of DH by retarding the diffusion of solvent and drug with this oil addition [19]. Additionally, Eudragit RS in situ forming gel prepared using NMP and incorporating peppermint oil (PO) (2.5–5% *w*/*w*) reduced DH release with an increased amount of PO [18]. 

**Figure 7:**
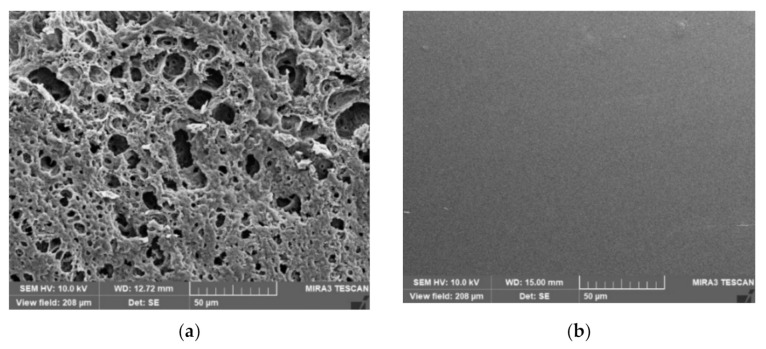
SEM images of DH-loaded rosin in situ forming gel after release test for ten days; (**a**) DHRL0 and (**b**) DHRL2.5 with 1000× magnification.

In this study, when the LO-added rosin in situ forming gel was introduced into the release medium, the prepared solution could not transform rapidly into the gel due to the barrier of oil retarding the penetration of the release medium into the system. Simultaneously, the hydrophilic drug diffused easily with DMSO into the release medium, causing a faster release rate in the oil-loaded formulation. The scanning electron microscopy (SEM) image (Figure 7) verified that the LO-loaded gel had a smooth surface and did not exhibit water penetration pores. The results were consistent with the previous report; the in situ forming gel prepared using a poly-(lactic acid) (PLA) polymer and a hydrophobic solvent, such as benzyl benzoate which showed a fast release rate compared with the small amount of hydrophilic solvent used [47]. Thus, an additive addition could affect the release rate depending on the type of polymer used [48]. The drug release rate could be controlled by a hydrophobic additive such as LO in rosin-based in situ forming gel systems. The rate of gel formation and the shape of the in situ forming gel are also factors considered to affect burst drug release.

### 2.6. Drug Release Mechanism

Different release kinetic models based on mathematical calculations were used to analyze the drug release behavior of DH-loaded rosin-based in situ forming gel systems. In this study, DH released profiles from four obtained formulations were fitted into four release models: zero order, first order, Higuchi’s, and the Korsmeyer–Peppas model. The Korsmeyer–Peppas model indicated the best correlation with the release data analysis of all formulations (Table 2). The r^2^ value closest to one indicates the best fit to the kinetic model. For the Korsmeyer–Peppas model, the type of diffusion is determined by the Fickian diffusion mechanism (n < 0.45), non-Fickian diffusion or anomalous (0.45 < n < 0.89), and matrix erosion (n > 0.89) [49]. The value of n in this study ranged from 0.383–0.455 for different oil-loaded formulations, suggesting the release of DH was mainly close to Fickian diffusion. Generally, a Fickian diffusion-driven matrix system release mechanism is involved with a concentration gradient, diffusion distance, and swelling degree [50]. For the DHRL0 formula, the value of n was obtained at 0.702, showing a non-Fickian diffusion mechanism. The release mechanism from this rosin-based in situ forming gel formulation was influenced by diffusion and erosion [51].

### 2.7. Scanning Electron Microscopy (SEM)

Figure 7 indicates SEM photos of LO-free and LO-loaded in situ forming gels after drug release test. The formulation without LO, DHRL0, had a sponge-like topography with an apparently porous surface (Figure 7a). The connected network promoted the DH diffusion from the solidified rosin matrix as a sustainable release pattern. The rapid phase separation and continuous polymeric matrix degradation caused the formation of large pores. It has been reported that the morphology of in situ forming gel, using Eudragit RS as the polymer, exhibited a porous surface, and the addition of hydrophilic polyethylene glycol (PEG 1500) promoted a more porous topography [52]. The number of pores is decreased when the solvent exchange rate is slow [53], whereas large pores are generated due to the rapid phase separation of the dissolved polymer [54].

Alternatively, the LO-loaded formulations, such as DHRL2.5, had a smooth, homogeneous surface with no pores (Figure 7b). DHRL5 and DHRL10 also had smooth surfaces (data not shown). The addition of oil could reduce the solvent exchange rate when the formulations were in contact with an aqueous buffer solution, causing a homogenous, softened, sticky gel and making them challenging to observe.

### 2.8. Antimicrobial Activities

The antimicrobial activities of drug-free rosin in situ forming gel, LO, DH solution in DMSO and DMSO are shown in Table 3a whereas those of in situ forming gel formulations containing DH (5%) with LO (0%, 2.5%, 5%, and 10%) are presented in Table 3b. These results were obtained from an assessment against various microorganisms using the agar-cup diffusion method. *Aggregatibacter actinomycetemcomitans* and *P. gingivalis* are the most common pathogens of periodontitis [2]. Microorganisms in the periodontal environment can cause gingival tissue inflammation and bone damage. Some bacterial species, including *S. aureus* and *E. coli*, are also found in periodontitis patients [2,55,56]. Therefore, these pathogens were examined for their antimicrobial activities in this study for the antimicrobial test. Table 3a shows the inhibition zone diameters of control groups. The DH-free in situ forming gel DRL0 showed weak antimicrobial activity against only *S. aureus* ATCC 43300 and *S. aureus* DMST 6532. A previous study indicated that the rosin acid and rosin acid-loaded nanoparticles showed antibacterial activities [57]. In this study, the viscous solution of the formulations formed a gel when placed on an agar surface, resulting in hardening the gel surface and retarding the diffusion of active compounds into the agar media. Although DMSO exhibits activity against tested microbes, it could not influence antimicrobial activity in rosin-based in situ forming gel formulations owing to its low potency and less diffusion. The control group which was DH dissolved in DMSO exhibited high inhibition against *A. actinomycetemcomitans* with a 41.7 mm clear zone diameter and moderate activity against other microbes with an inhibition zone diameter of >30 mm, except for *E. coli*. However, the inhibition zone diameter of DH-D against S. aureus groups was not much different from LO. LO displayed high activity on *S. aureus* ATCC 6538 and *A. actinomycetemcomitans*. The *S. aureus* and methicillin-resistant *S. aureus* (MRSA) are found in the oral cavity [58,59]. Patients with gingivitis-periodontitis have a prospective reservoir of opportunistic pathogens such as *S. aureus* in their oral cavity and subgingival pockets [60]. The susceptibility of *S. aureus* and *MRSA* strain (ATCC 43300) to LO is beneficial for use in the treatment of oral cavity disease.

All drug-loaded formulations showed antimicrobial activities against the tested bacterial strains (Table 3b). All formulations exhibited good activity against *P*. *gingivalis* and *S*. *aureus* groups, while a weak inhibition was observed against *A. actinomycetemcomitans* and *E*. *coli*. They efficiently inhibited a methicillin-resistant *S. aureus* (MRSA) strain such as *S. aureus* (ATCC 43300). The 2.5% and 5% LO-loaded formulations exhibited nearly the same inhibition zone diameter and showed fewer antimicrobial activities than DHRL0 towards the tested microbes. The 10% LO concentration increased the antimicrobial activities of *P*. *gingivalis* and *S*. *aureus* groups compared to 2.5% and 5% LO loading (*p* < 0.05). The LO-loaded formulations had weak activity against *E. coli* since LO had low activity against this strain, and corresponded with the previous report indicating LO more effective in inhibiting *S. aureus* than *E.* *coli* [61]. The cell membranes of Gram-negative bacteria contain a more complex lipopolysaccharide and phospholipid layer with a significantly lower diffusion rate than the lipophilic-based antimicrobial compounds of the essential oils [62]. Nevertheless, the nanoemulsions with LO incorporation exhibited similar antimicrobial activity against bacteria, including *S*. *aureus*, *E*. *coli*, and *Salmonella* spp. [63]. For antibiotic susceptibility, the minimum inhibitory concentrations (MICs) of DH against *S. aureus*, *E. coli*, *P. gingivalis* and *A. actinomycetemcomitans* are 3, 1, 0.5, 2.1 μg/mL, respectively [64,65,66]. In practice, the volume of gingival crevicular fluid in the periodontal pocket of patients with periodontitis is 5–20 μL [67]. When 10 μL of formulation was injected into this pocket with 20 μL gingival crevicular fluid, the amount of DH released as shown in Figure 6b from in situ forming gels was more than 50% or more than 8333 μg/mL, which is apparently above the MIC against all test microbes. The commercial product such as Atridox also contains 5 or 10% DH loading for periodontitis treatment [9].

Our results indicate that the DH-loaded formulation showed promising antimicrobial effects against *S. aureus* groups, *P. gingivalis,* and *A. actinomycetemcomitans.* The LO addition to the in situ forming gel formulation enhanced the antimicrobial activity of DH. Thus, lower LO concentrations did not improve the antimicrobial activities of loaded drugs. However, 10% LO concentration showed high antimicrobial activities against *P. gingivalis* and *S. aureus* ATCC 6538. The synergistic effect has been reported for doxycycline with terpene components of essential oils encapsulated in lipid nanocapsules against Gram-negative strains [26]. Therefore, the high LO content and DH might synergise the antibacterial activities against tested microbes. The other major component of volatile oil such as carvacrol (oregano oil), eugenol (clove oil) and cinnamaldehyde (cinnamon oil) should be further investigated for loading into developed formulations since there was synergistic effect with a combination of doxycycline for antimicrobial activities [26]. In addition, the combination of DH and metronidazole, which exhibited synergy against anaerobic pathogens such as *P. gingivalis* [68], are also interesting for use as the active compounds for periodontitis treatment by incorporation in an in situ forming gel.

## 3. Conclusions

This study prepared a DH-loaded rosin-based in situ forming gel with LO. Then, based on gel formation features, LO concentrations (2.5, 5, and 10%) were selected for incorporation into the rosin-based in situ forming gel using DMSO as a solvent. The initial gel formation rate and interfacial interaction phenomena of the in situ forming gel could be observed clearly under an inverted microscope. The LO addition slightly increased viscosity and injectability. The hardness of the gels was weakened by LO addition due to its retardation of water diffusion into the system. The release profiles of DH from all formulations showed a sustained release over ten days. It was discovered that the addition of LO influenced the release of DH. Diffusion was the main release mechanism for LO-added in situ forming gel. All DH-loaded samples showed the inhibition zone diameter against tested microbes including pathogens of periodontitis according to the antimicrobial test. The 10% LO-loaded in situ forming gel promoted antimicrobial properties. LO showed strong antimicrobial activities on the tested strains, especially on the *S. aureus* group and *P. gingivalis*. The findings revealed that incorporating LO into the rosin in situ forming gel can modify drug release rates and promote the antimicrobial activities of periodontal delivery systems for periodontitis treatment. The incorporation of other volatile oils comprising synergist activity with the combination of an antimicrobial drug in an injectable rosin-based in situ forming gel should be further investigated.

## 4. Materials and Methods

### 4.1. Materials

Rosin was obtained from Karnchanapon Co. Ltd., Nakhon Pathom, Thailand. LO was procured from Thai-China Flavours and Fragrances Industry Co., Ltd., Patumthani, Thailand. NMP (Lot No. A0251390, Fluka, New Jersey, USA) and DMSO (Lot No. 453035, Fluka, Buchs, Switzerland) were employed as the solvents. DH (Huashu Pharmaceutical Corporation, Shijiazhuang, China) was used as model drug. Agarose (Lot No. H7014714, Vivantis, Selangor Darul Ehsan, Malaysia) was used to analyze the gel formation behavior under inverted microscope. Potassium dihydrogen orthophosphate (Ajax Finechem, New South Wales, Australia) and sodium hydroxide (Ajax Finechem, New South Wales, Australia) were used to prepare phosphate buffer solution (PBS; pH = 7.4). Sheep blood and chocolate agar (Ministry of public health, Thailand), tryptic soy agar, and tryptic soy broth (DifcoTM, Detroit, MI, USA) were used as media for antimicrobial testing.

### 4.2. Preparation of In Situ Forming Gel 

Rosin (55% *w*/*w*) was dissolved in DMSO and NMP solvents. Then, different LO concentrations (1, 2.5, 5, 10, 15, and 20% *w*/*w*) were incorporated into the prepared solutions and mixed vigorously using a magnetic stirrer at room temperature. The accurate weight of DH was added to the selected formulation and stirred until completely dissolved. The components of drug-free and DH-loaded in situ forming gel formulations with different LO concentrations are shown in Table 4.

### 4.3. Study of Gel Formation

#### 4.3.1. Gel Formation

The morphology of gel formation for drug-free rosin-based in situ forming gel systems containing different concentrations of LO in NMP/DMSO after injecting the solutions through a 1 mL syringe into PBS (pH 7.4) was recorded to observe during the initial period of the solvent diffusion and gel formation process. The photo was taken at a predetermined time after the transformation of the solution into the gel layer.

#### 4.3.2. Microscopic Observation of Gel Formation

The prepared formulations were tested for their phase transition processes in cross-sectional view under a stereomicroscope (Motic Asia, Kowloon, Hong Kong). The 0.6% *w*/*w* agarose solution was prepared by dissolving in heated PBS pH 7.4 at 60 °C, and the solution was poured into petri dishes (diameter: 4.5 cm). The agarose gel was made into a well with a stainless-steel cylinder cup (diameter: 6 mm) and filled with 150 µL prepared in situ forming gel formulations. Gel formation as an opaque ring surrounding the agarose rim was recorded under a stereoscope at different time intervals. 

#### 4.3.3. Interfacial Phenomenon

The interface interaction between aqueous phase and in situ forming gel formulations at the initial phase was investigated. The 0.6% *w*/*w* agarose was prepared as mentioned above, and the solution was poured onto a glass slide. Then, near the edge of the settled agarose gel, 50 µL formulation was dropped using a micropipette. The interface interaction after solvent exchange was observed by capturing the image at different time intervals under stereomicroscope (TE-2000U, Nikon, Kaw, Japan).

### 4.4. Viscosity and Injectability

The viscosity of drug-free and drug-loaded in situ forming gel systems was measured using a viscometer (Brookfield Engineering Laboratories Inc., Middleborough, MA, USA) at 25 °C at various shear rates. A 0.5 mL sample was put on a cone plate, and the viscosity was recorded. The measurement was done in triplicate. The ease of injecting formulations was investigated using a texture analyzer (TAXT plus, Stable Micro Systems, Godalming, UK) in a compression mode, using a 1 mL syringe and 24G needle, which was clamped to a stainless-steel stand for analysis. The upper probe of the instrument was forced downward at a constant speed (1.0 mms^−1^), and a force of 0.1 N was compressed to the syringe barrel base. Injectability was presented as a force (N) or work of injection (N.mm). The experiments were conducted in triplicate.

### 4.5. Mechanical Properties Study

The mechanical properties, including hardness and adhesion of the prepared in situ forming gels, were determined using a texture analyzer (TAXT plus, Stable Micro Systems, Godalming, UK). The drug-loaded in situ forming gel formulations were selected to determine mechanical features. Agarose gel 0.6% *w*/*v* was prepared in petri dish as mentioned above. The prepared 0.6% *w*/*w* agarose was made into a hole to fill formulations of 150 µL, and the gel formation was left for 74 h for complete phase inversion. Then, the obtained gels of all formulations were measured for their hardness and adhesiveness using a downward force of 5 g, which was applied for 60 s to ensure contact between the probe and gel surface. The probe was lifted at a speed of 0.5 mm/s to a predefined distance of 5 mm and the established force–distance curve for each formulation was recorded. The hardness was measured as the maximum deformation force needed to penetrate the probe into the obtained gel, and the adhesion was denoted as the force needed to detach the probe from the gel surface [20]. The measurements were performed in triplicate.

### 4.6. Wettability Study

The wettability of rosin-based in situ forming gel containing LO (0, 2.5, 5, and 10%) and DH was assessed using a contact angle measurement method using goniometer (FTA 1000, First Ten Angstroms, Newark, CA, USA). The measurement was done at five seconds after dropping the formulation on the glass slide and the agarose gel surface. The measurements were conducted in triplicate.

### 4.7. In Vitro Drug Release Studies

The formulations formed gel after injecting 10 mL PBS pH 7.4 into a 25 mL vial and being kept in a shaking incubator at 37 °C with mild rotation shaking at 50 rpm. The weight number of gel solutions at 0.03 g were used for release studies. The DH content of all formulations was determined before drug release test using UV–Vis spectrophotometer (Cary 60 UV-Vis, Model G6860A, Agilent, Santa Clara, CA, USA) at 347 nm (n = 6). At a predetermined time, the aliquot of 2 mL released sample was withdrawn, and an equal amount of fresh medium was replaced to maintain the sink conditions. The release of DH was determined using UV–Vis spectrophotometer at 347 nm. (n = 3). The dissolution data were fitted to mathematical equations such as zero order, first order, Higuchi’s and Korsmeyer–Peppas models. The n-value from the Korsmeyer–Peppas equation was applied to determine the mechanism of drug release. The DD-Solver software application, an add-in program for Microsoft Excel, was used to determine the release mechanism. 

### 4.8. Scanning Electron Microscopy (SEM)

After the drug release test, the gel was dried using the freeze dryer (Triad Labconco, MI, USA) and kept in a desiccator until analysis. The scanning electron microscope (SEM) (TESCAN MIRA3, Brno-Kohoutovice, Czech Republic) was used for determination of the surface topography of dried samples at an accelerating voltage of 15 kV.

### 4.9. Antimicrobial Activity Study

The DH-loaded formulation with different LO concentrations, drug-free rosin in situ forming gel, and control groups (LO, DMSO, and 5% DH in DMSO) were investigated against *S*. *aureus* ATCC 6538, *S*. *aureus* ATCC 43300, *S*. *aureus* DMST 6532, *S*. *aureus* ATCC 25923, *E. coli* ATCC 8739, *A*. *actinomycetemcomitans* and *P*. *gingivalis*. The antimicrobial activities were determined using the agar-cup diffusion method. The bacteria inocula were incubated for 36 h in tryptic soy broth and the turbidity of broth suspensions of organisms was checked using 0.5 McFarland standard. Then, the prepared broth suspensions of *S. aureus* ATCC 6538, *S*. *aureus* ATCC 43300, *S*. *aureus* DMST 6532, *S*. *aureus* ATCC 25923, *E. coli* ATCC 8739 were swab-spread on the tryptic soy agar plates, whereas sheep blood and chocolate agar were used as media for antimicrobial testing of *P. gingivalis* and *A. actinomycetemcomitans,* respectively. The sterilized cylinder cups (8 mm in diameter and 10 mm in height) were carefully placed on the swabbed agar surface. The 150 L prepared formulations and samples were filled into these cylinder cups and incubated for 48 h at 37 °C. An anaerobic incubator (Forma Anaerobic System, Thermo Scientific, Ohio, USA) was used for incubating anaerobic bacteria (*A*. *actinomycetemcomitans* and *P*. *gingivalis*). The diameter (mm) of the inhibition zone (n = 3) was used to indicate the antimicrobial activities.

### 4.10. Statistical Analysis

Statistical significance of all data was examined using the one-way analysis of variance followed by the Tukey test. The significance level was set at *p* < 0.05. The analysis was conducted using SPSS for Windows (version 11.5).

## Figures and Tables

**Table 1:** Physical properties of drug-free/drug-loaded rosin/LO in situ forming gel systems. Results are shown as mean values ± SD, (n = 3).

Formula	Viscosity (cP)	Injectability (24 Gauge Needle)	
		Force (N)	Work (N.mm)
NRL0	191.40 ± 0.36 ^a^	4.05 ± 0.13 ^a^	69.57 ± 0.98
NRL1	172.23 ± 1.2 ^b^	3.53 ± 0.49 ^ab^	59.42 ± 2.78
NRL2.5	169.13 ± 0.93 ^c^	3.46 ± 0.14 ^b^	60.84 ± 0.64
NRL5	136.67 ± 1.62 ^d^	2.81 ± 0.13 ^c^	51.36 ± 3.49
NRL10	140.30 ± 0.72 ^e^	2.81 ± 0.17 ^c^	51.95 ± 2.07
NRL15	140.73 ± 1.00 ^e^	2.92 ± 0.16 ^c^	52.05 ± 1.60
NRL20	143.37 ± 1.32 ^f^	2.78 ± 0.13 ^c^	51.94 ± 0.22
DRL0	177.57 ± 2.93 ^a^	3.00 ± 0.29 ^a^	52.76 ± 1.02
DRL1	157.61 ± 2.02 ^b^	2.52 ± 0.18 ^ab^	45.87 ± 2.14
DRL2.5	158.20 ± 1.15 ^b^	2.58 ± 0.13 ^ab^	46.89 ± 1.92
DRL5	141.80 ± 2.75 ^c^	2.57 ± 0.19 ^ab^	46.19 ± 2.23
DRL10	134.17 ± 2.37 ^d^	2.41 ± 0.22 ^b^	45.21 ± 2.57
DRL15	124.37 ± 0.64 ^e^	2.37 ± 0.13 ^b^	43.09 ± 2.10
DRL20	122.60 ± 0.26 ^e^	2.42 ± 0.08 ^b^	43.76 ± 1.37
DH-RL0	624.51 ± 10.39 ^a^	14.7 ± 1.58 ^a^	258.2 ± 32.76
DH-RL2.5	644.09 ± 3.25 ^a^	14.90 ± 0.12 ^a^	262.84 ± 12.52
DH-RL5	665.94 ± 10.16 ^ab^	17.81 ± 0.50 ^b^	315.69 ± 16.40
DH-RL10	684.15 ± 11.85 ^bc^	21.47 ± 1.48 ^c^	359.69 ± 30.68

The superscripts (^a–f^) in the column represent a significant difference within the tested formulations (*p <* 0.05). (No superscript) not determined, DH = doxycycline hyclate.

**Table 2:** The regression coefficient (r^2^) value and diffusion exponent value (n) obtained from the different model fitting of the release profile of DH-loaded rosin in situ forming gel systems.

Formula	Zero Order	First Order	Higuchi’s	Korsmeyer–Peppas		
	r^2^	r^2^	r^2^	r^2^	n	Release Mechanism
DHRL0	0.9298	0.964	0.9475	0.9945	0.702	Non-Fickian diffusion
DHRL2.5	0.5788	0.7833	0.9194	0.9213	0.455	Fickian diffusion
DHRL5	0.5995	0.7912	0.9538	0.969	0.407	Fickian diffusion
DHRL10	0.5299	0.8333	0.9202	0.9452	0.383	Fickian diffusion

**Table 3:** Inhibition zone diameters (mm) against tested microorganisms; (a) control groups of drug-free-RL0, LO, DH in DMSO (D) and DMSO solvent; (b) different in situ forming gel formulations of 5% DH and 0, 2.5, 5, and 10% LO. Results are indicated as mean values ± SD, (n = 3).

(a)				
Microorganisms	Inhibition zone diameter (mm.) (mean ± S.D.)			
	DRL0	Lime peel oil	DH-D	DMSO
*S. aureus* ATCC 6538	-	40.3 ± 2.5 ^b^	31.3 ± 0.6 ^c^	11.7 ± 0.6 ^d^
*S. aureus* ATCC 43300	11.0 ± 1.0 ^a^	31.0 ± 1.7 ^b^	30.7 ± 0.6 ^b^	12.7 ± 1.2 ^a^
*S. aureus* DMST 6532	10.0 ± 0.0 ^a^	32.3 ± 1.5 ^b^	32.0 ± 1.6 ^b^	14.0 ± 1.0 ^a^
*S. aureus* ATCC 25923	-	34.0 ± 1.7 ^b^	31.0 ± 1.0 ^b^	12.3 ± 0.6 ^c^
*P* *. gingivalis*	-	15.3 ± 1.0 ^b^	31.3 ± 1.5 ^c^	14.0 ± 1.0 ^b^
*A* *. actinomycetemcomitans*	-	36.0 ± 1.5 ^b^	41.7 ± 1.5 ^c^	24.0 ± 1.5 ^d^
*E. coli* ATCC 8739	-	14.0 ± 1.0 ^b^	24.7 ± 1.5 ^c^	11.3 ± 1.2 ^b^
(b)				
Microorganisms	Inhibition zone diameter (mm.) (mean ± S.D.)			
	DHRL0	DHRL2.5	DHRL5	DHRL10
*S. aureus* (ATCC 6538)	19.0 ± 1.0 ^a^	17.7 ± 1.2 ^a^	17.7 ± 0.6 ^a^	21.7 ± 0.6 ^b^
*S. aureus* (ATCC 43300)	27.0 ± 0.6 ^a^	21.3 ± 1.2 ^b^	21.7 ± 0.6 ^b^	28.0 ± 1.0 ^a^
*S. aureus* (DMST 6532)	28.0 ± 1.0 ^a^	20.0 ± 1.0 ^b^	21.7 ± 1.2 ^b^	29.3 ± 0.6 ^a^
*S. aureus* (ATCC 25923)	27.3 ± 0.6 ^a^	20.7 ± 1.5 ^b^	21.3 ± 1.5 ^b^	25.0 ± 1.0 ^a^
*P* *. gingivalis*	22.3 ± 1.5 ^a^	22.3 ± 2.1 ^a^	21.0 ± 1.0 ^a^	26.0 ± 2.6 ^b^
*A* *. actinomycetemcomitans*	18.0 ± 0.0 ^a^	15.7 ± 0.6 ^b^	15.7 ± 0.6 ^b^	17.0 ± 1.0 ^ab^
*E. coli* ATCC 8739	15.3 ± 0.6 ^a^	11.7 ± 0.6 ^bc^	10.7 ± 0.7 ^b^	12.7 ± 0.6 ^c^

The superscripts (^a–d^) in the rows represent a significant difference within the tested formulations (*p* < 0.05). (-) no inhibition zones.

**Table 4:** The composition of drug-free and DH-loaded in situ forming gel formulations with different LO concentrations.

Formula	DH (%*w*/*w*)	LO (%*w*/*w*)	Rosin (%*w*/*w*)	NMP (%*w*/*w*)	DMSO (%*w*/*w*)
NRL0	-	0	55	45	-
NRL1	-	1	55	44	-
NRL2.5	-	2.5	55	42.5	-
NRL5	-	5	55	40	-
NRL10	-	10	55	35	-
NRL15	-	15	55	30	-
NRL20	-	20	55	25	-
DRL0	-	0	55	-	45
DRL1	-	1	55	-	44
DRL2.5	-	2.5	55	-	42.5
DRL5	-	5	55	-	40
DRL10	-	10	55	-	35
DRL15	-	15	55	-	30
DRL20	-	20	55	-	25
DHRL0	5	0	55	40	-
DHRL2.5	5	2.5	55	37.5	-
DHRL5	5	5	55	35	-
DHRL10	5	10	55	30	-

## Data Availability

The data presented in this study are available on request from the corresponding author.
